# The new fitness world: commodifying well-being in the neoliberal era

**DOI:** 10.31744/einstein_journal/2026RW1778

**Published:** 2025-12-04

**Authors:** Bruno Gualano, Hamilton Roschel, Antonio Valverde

**Affiliations:** 1 Universidade de São Paulo Center of Lifestyle Medicine Faculdade de Medicina São Paulo SP Brazil Center of Lifestyle Medicine, Faculdade de Medicina, Universidade de São Paulo, São Paulo, SP, Brazil.; 2 Pontifícia Universidade Católica de São Paulo São Paulo SP Brazil Pontifícia Universidade Católica de São Paulo, São Paulo, SP, Brazil.

**Keywords:** Physical fitness, Well-being, Neoliberalism, Health policies

## Abstract

This paper examines the fitness industry as a by-product of neoliberal ideology, using *"The New Way of the World: On Neoliberal Society"* as a conceptual framework. Neoliberalism, as articulated by Dardot and Laval, extends beyond economic policy to shape governance and human behavior by embedding market-driven rationality into everyday life. The fitness industry, with its emphasis on individual responsibility for health and self-optimization, exemplifies core neoliberal tenets. It fosters the notion of the "entrepreneurial self," wherein success or failure is attributed to personal merit, thereby marginalizing collective health initiatives. The proliferation of fitness influencers further reinforces this paradigm, often disseminating misinformation and promoting unrealistic ideals of health and fitness. We argue that the commodification of health within the fitness industry aligns with neoliberalism's foundational principles: competitiveness, self-regulation, and the erosion of collective structures. In response, we advocate for a shift toward health frameworks rooted in inclusivity, equity, and shared responsibility, challenging the individualistic ethos embedded in current fitness discourse. Reimagining health policy through a lens of solidarity offers a pathway to dismantling neoliberal rationality and fostering a more democratic and equitable health system.

## INTRODUCTION

### The fitness world as an expression of neoliberal rationality

"‘We are the champions’ – such is the hymn of the new entrepreneurial subject. From the song's lyrics, which in their way heralded the new subjective course, the following warning in particular must be retained: ‘No time for losers.’ What is new is precisely that the loser is the ordinary man, the one who in essence loses." (Dardot and Laval, 2016, p. 356)^(1)^

Recent work in the sociology of the body, such as Singh's^(2)^ ethnographic study of Muay Thai and kickboxing practitioners in East London, highlights how bodily practices may function as sites of ethical and existential reorientation within a neoliberal framework. In Singh's account, combat training offers a rule-bound, structured environment through which individuals respond to conditions of precarity and exclusion, reconstructing selfhood and reasserting agency. While this stands in contrast to the commodified fitness culture examined here, where the body is disciplined according to market logics of productivity and self-optimization, the comparison remains philosophically instructive. Both contexts illustrate how neoliberal rationality organizes bodily life, positioning the body as a medium through which subjectivity is constituted. Whether through compliance or contingent resistance, the body becomes the site through which individuals negotiate their place in a world governed by the imperative of self-entrepreneurship.

The "fitness world," also referred to as the fitness industry or fitness culture, serves as an umbrella term encompassing a broad range of activities, trends, and practices ostensibly aimed at promoting health, fitness, and wellness. Its underlying philosophy centers on the pursuit of a "healthy" body and ideal physique, typically framed through discourses of balanced diets and active lifestyles. However, the fitness world also reveals itself as a commercial enterprise, wherein the ultimate goal is the sale of products and services designed to achieve these aesthetic and health ideals.

Framed within the pursuit of idealized standards of beauty and performance, the fitness industry offers numerous forms of human enhancement.^*^ These include anabolic steroids, cosmetic surgery, nutritional supplements, cosmetics, spas, saunas, massage therapy, "holistic" treatments, meditation apps, digital platforms, wearable fitness trackers, exercise equipment, gym memberships, and private or group training sessions. The market also encompasses trade fairs and professional development courses for fitness professionals. In essence, anything that can be commodified and marketed under the promise of promoting health and well-being, regardless of its actual efficacy,^**^ falls within the purview of this industry.

The expansion of the fitness world has been significantly amplified by the emergence of fitness influencers: individuals who model idealized bodies and lifestyles on social media and actively drive consumer engagement across the industry. While many of these influencers position themselves as advocates of science-based health practices, the content they produce frequently lacks scientific rigor.

A recent study assessed the qualifications of leading Brazilian fitness influencers, defined as those with over 100,000 Instagram followers, and evaluated the scientific quality of their posts.^(4)^ The findings revealed that (a) one in four influencers had no academic training; (b) those with fewer academic credentials often had larger follower counts; and (c) there was no significant correlation between follower count and post quality.

Qualitative analysis further showed that the overall quality of the posts was low (39±26%), with only ∼2.7% of posts (13 in total) citing any supporting references. Based on these data, the authors concluded that prominent Brazilian fitness influencers disseminate low-quality information on exercise and health, contributing to the widespread circulation of misinformation among millions of followers.

This study is instrumental in dispelling the assumption that fitness influencers operate under scientific guidance. In reality, the key figures within the fitness industry function within a neoliberal framework, a theme explored in greater depth throughout this paper.

In *"The New Way of the World: On Neoliberal Society"*,^(1)^ Dardot & Laval argue that neoliberalism extends beyond economic doctrine to operate as a rationality that organizes governance and human conduct. It permeates daily life by encouraging individuals to function as "entrepreneurs of themselves." Table 1 outlines the principal philosophical tenets of neoliberalism as articulated by these authors.

**Table 1:** Philosophical principles of neoliberalism in The New Way of the World: On Neoliberal Society^(1)^

Philosophical principle	Description
Principle of Governmentality	Neoliberalism is conceived as a form of governmentality that transcends institutional structures, shaping individual behavior according to market-based rationality
Competitiveness as a Universal Standard	Competitiveness is upheld as a core societal value, with individuals compelled to act as self-optimizing agents continually striving for peak performance
Resignification of Freedom	Freedom is redefined in market terms: individuals are considered free when they compete and self-regulate, independent of collective autonomy or the public good
Utilitarianism and Rational Calculation	Well-being is pursued through rational cost–benefit calculations, with the market positioned as the most efficient mechanism for social organization
Dissolution of the Collective	Neoliberalism erodes collective structures by privatizing responsibilities once managed collectively, such as health, education, and welfare
The Spontaneous Order of the Market	Influenced by Hayek,^***^ this principle holds that the market constitutes a natural, self-regulating order capable of organizing resources and behavior without state oversight

Neoliberalism also promotes the notion that competition is the fundamental organizing principle of society. This engenders an individualistic ethic in which success and failure are framed as reflections of personal merit. As Dardot & Laval observe:

*"…neoliberal rationality produces the subject it requires by deploying the means of governing him so that he really does conduct himself as an entity in a competition, who must maximize his results by exposing himself to risks and taking full responsibility for possible failures. ‘Enterprise’ is thus the name to be given to self-government in the neoliberal age. This ‘entrepreneurial self-government’ is something other, and much more, than the ‘enterprise culture’ referred to above." (Dardot & Laval, 2016, p. 328)* ^(1)^

### The "Entrepreneurial Self" and the Myth of Meritocratic Bodies

A brief examination of common slogans and discourses within the fitness world reveals their deep entrenchment in neoliberal ideology. Consider, for instance, the widely circulated phrase "no pain, no gain." Despite being repeatedly refuted by scientific research, this slogan encapsulates the neoliberal ideal that success necessitates sacrifice. The individual is expected to forgo pleasure and devote personal effort toward self-improvement, reinforcing a meritocratic ethic in which self-sufficiency and personal discipline are construed as the only legitimate routes to achievement.

This logic reflects the broader neoliberal conceptualization of the individual as an entrepreneur of the self, a notion in which "self-care" becomes analogous to managing one's personal capital, as proposed by Dardot and Laval. Within this framework, fitness influencers, coaches, and related figures find fertile ground. They amplify the ideology of self-improvement by promoting the body as a site of continuous investment, optimization, and branding. The body is thus reimagined as a personal enterprise, subject to design, style, and performance, naturalizing the obligation to enhance productivity and remain competitive.

The emergent discourse of pleasure and performance, which disrupts prior social norms, demands a new kind of "ideal body," one that exceeds previous capacities for both production and enjoyment. As Dardot and Laval argue, neoliberalism elevates the winner over the ordinary individual, who is implicitly cast as the "loser." This construction mirrors the emergence of a new subject: one primed for competition, performance, and ultimately, success. Featherstone^(5)^ further elaborates on this phenomenon by conceptualizing the neoliberal "ideal body" not merely as a visual ideal but as an affective instrument. Success in fitness culture entails not only aesthetic conformity but also the ability to project curated affective intensities, charisma, discipline, and desirability. Featherstone's distinction between the static "body image" (a mental representation rooted in visual appearance) and the dynamic "body without image" (a medium of embodied affect communicated through movement, posture, and gesture) is particularly instructive. In this context, the "body without image" may surpass surgically modified aesthetics by signaling vitality and presence through embodied performance. Within the neoliberal logic of the fitness industry, both dimensions are commodified: the body image is curated for visual consumption, while the affective output of the "body without image" serves social performance. Together, they reinforce new hierarchies, not only through visible markers, but through embodied charisma and perceived energy. The individual is thus transformed into a product in perpetual optimization, managing both physical form and affective labor to remain competitive, fully embodying Dardot and Laval's concept of the entrepreneurial self. Another neoliberal maxim circulating within fitness culture is the triad "focus, strength, and faith."^****^

This slogan similarly reinforces the individualization of success, functioning as a secular extension of prosperity theology^(6)^ applied to the human body. It suggests that bodily perfection and superior performance are attainable through sheer will and personal dedication. This discourse implicitly pathologizes structural barriers. For instance, when a Black, working-class, single parent from the urban periphery lacks the time, energy, or resources to engage in regular exercise or prepare nutritionally balanced meals, neoliberal logic reduces this systemic issue to a personal failure, because, in this worldview, competitiveness is universal, and success is strictly a matter of individual resolve.

This framing is not only alienating and exclusionary, but also empirically flawed. It disregards a substantial body of evidence demonstrating that health behaviors are significantly influenced by socioeconomic, cultural, and structural factors.^(7-9)^ Nevertheless, under neoliberal ideology, failure, whether in health, finance, or social standing, is reframed as a lack of personal effort or insufficient sacrifice. Such individualization obscures the root causes of inequality and sustains a model of health that privileges autonomy over equity. In aligning itself with these neoliberal principles, the fitness world contributes to the erosion of collective responsibility and public health infrastructure. By valorizing self-governance and entrepreneurial attitudes, it reinforces the privatization of health and the marginalization of those who lack access to its commodified forms. This dynamic underscores the urgent need to reimagine health beyond market logic. A more equitable and democratic health framework must prioritize inclusivity, solidarity, and shared responsibility, challenging the individualistic ethos that underpins contemporary fitness culture.

### Pathways Toward Collective and Solidary Health

For Dardot and collaborators, neoliberalism is not merely an economic doctrine but a totalizing rationality that reshapes how individuals think, act, and relate to one another. It permeates nearly all domains of social life, organizing behavior around competition, self-interest, and market logic. Overcoming this dominance, they argue, requires the invention of a new form of governmentality rooted in collective praxis, what they term the reason of the commons.^*****^ This alternative framework challenges the entrepreneurial self by fostering "counter-conducts": practices of solidarity and democratic participation that resist market-driven individualism.

Public initiatives such as Brazil's Food Guide for the Brazilian Population and the Health Academy Program exemplify institutional counter-conducts. These programs promote inclusive, community-based approaches to health that prioritize structural interventions over individualized behavior change. Beyond state-led efforts, grassroots innovations, such as food cooperatives^******^ and worker-owned wellness spaces, embody principles of shared governance and collective care. Through democratic ownership, sliding-scale access, and reinvestment into local initiatives, these efforts reframe health not as a commodity but as a shared right and resource.

Despite these advances, such models remain constrained by dominant cultural narratives that equate health with personal responsibility and aesthetic performance. These narratives obscure structural determinants of health and reinforce exclusion, particularly for marginalized populations. The commodification of health, as typified by the fitness industry, transforms wellness into a luxury good, exacerbating inequities and individualizing risk. For individuals living in precarious socioeconomic conditions, this model often offers little more than moral judgment masquerading as empowerment.

Dismantling this paradigm requires more than policy reform; it demands a cultural and epistemological shift. A promising avenue lies in the transformation of health education. By integrating themes such as social justice, critical thinking, and the social determinants of health into professional training, academic institutions can cultivate a generation of health practitioners equipped to navigate and challenge the complexities of contemporary care. These professionals would not only treat individuals but also advocate for systemic equity and structural change.

Ultimately, advancing collective and solidary health requires a redefinition of success, from individual achievement to communal well-being. This reorientation entails resisting entrenched norms of competition, self-optimization, and aesthetic judgment in favor of mutual support, inclusivity, and public accountability. As Dardot & Laval assert, rejecting the market as the dominant model of social organization opens space to imagine a truly democratic and inclusive health system, one that prioritizes care, dignity, and equity over profit.

This vision is echoed by Hallal et al.,^(11)^ who emphasize the role of public policy in enabling physical activity as a societal norm. They write:

*"Governments are responsible for making [physical activity] easy for individuals to choose […] as part of routine living. Access to pleasant, safe, healthy, equitable and purposeful physical activity must be a societal priority." (Hallal et al., 2024, p. 2)*

Table 2 illustrates how the fitness world embodies core tenets of neoliberal rationality, competition, individual calculation, and the erosion of collective structures, underscoring the urgency of constructing alternative health frameworks. Achieving this transformation will require coordinated efforts across sectors, including academia, government, civil society, and local communities. Together, these actors can contest neoliberal ideology and build infrastructures of care that are inclusive, accessible, and rooted in solidarity. In doing so, they lay the foundation for a health system grounded not in consumption and exclusion, but in shared humanity and collective responsibility.

**Table 2:** The fitness world through the lens of neoliberal principles, as outlined in The New Way of the World: On Neoliberal Society^(1)^

**Competitiveness as a Universal Standard**
The fitness world promotes the belief that success stems from individual effort, reinforcing the ideal of the "entrepreneurial self." Slogans such as "no pain, no gain" and "focus, strength, and faith" exemplify this ethos, linking sacrifice, motivation, and merit to physical transformation
**Principle of Governmentality**
The fitness world illustrates how neoliberalism governs conduct through market rationality, encouraging individuals to pursue optimal aesthetics and physical performance as personal obligations
**Resignification of Freedom**
Freedom is redefined as self-regulation and consumer autonomy, wherein individuals "invest" in their bodies according to prevailing norms of appearance and performance
**Utilitarianism and Rational Calculation**
Decisions regarding health and aesthetics are framed as rational investments aimed at maximizing personal benefit, with collective or structural determinants largely ignored
**Dissolution of the Collective**
Health and well-being are construed as individual responsibilities, independent of social or environmental contexts. Collective mechanisms, such as public health policies, are devalued in favor of personalized, market-driven solutions
**Market Naturalization**
Fitness products and services are portrayed as indispensable for achieving health and wellness, thereby normalizing bodily commodification and dependency on market-based interventions

## Data Availability

The underlying content is contained within the manuscript.

## Footnotes

* Human enhancement refers to "any kind of genetic, biomedical, or pharmaceutical intervention aimed at improving human dispositions, capacities, or well-being, even if there is no pathology to be treated."^(3)^

** Many practices associated with the pursuit of an idealized body are known to entail significant health risks. These range from psychological and behavioral disorders, such as mood disturbances, body image dissatisfaction, and disordered eating, to more severe outcomes, including mortality. The latter has become increasingly common among individuals who abuse anabolic steroids, misuse other enhancement drugs, or undergo invasive aesthetic procedures. Contrary to popular belief, the fitness industry cannot be said to unequivocally promote health and well-being through its products and services.

*** Friedrich Hayek (1899–1992), an Austrian economist and philosopher, was a prominent proponent of classical liberalism and the market economy. He argued that economic freedom is essential for societal prosperity and maintained that state intervention disrupts the natural social order. Hayek is also well known for advancing the concept of "spontaneous order," the idea that markets and societies function more effectively in the absence of centralized control.

**** The phrase Focus, Strength, and Faith is a widely used slogan in Brazil, symbolizing inspiration, determination, and motivation, and ultimately reinforcing the ideal of persistence in pursuit of one's "dream."

***** Dardot and Laval formally define counter-conduct as "practices of ‘communization’ of knowledge, mutual aid, and cooperative work [that] can delineate the features of a different world reason."

****** A concrete example of such counter-conduct is provided by the Landless Workers’ Movement (*Movimento dos Trabalhadores Rurais Sem Terra*, MST) in Brazil, which organizes agroecological cooperatives that operate under collective ownership. These cooperatives produce organic food, such as large-scale rice crops, through sustainable methods, offering a direct challenge to agribusiness and commodified food systems.^10^

## References

[1] Dardot P, Laval C (2016). Echalar M, tradutor.

[2] Singh A (2023). Learning to labour in the gym: Training to fight to reimagine the self and work under neoliberalism. Sociology Compass.

[3] Giubilini A, Sanyal S (2016). The ethics of human enhancement: understanding the debate.

[4] Marocolo M, Marocolo IC, da Mota GR, Simim MA, Franchini E, Costa VT (2021). Is social media spreading misinformation on exercise and health in Brazil?. Int J Environ Res Public Health.

[5] Featherstone M (2010). Body, image and affect in consumer culture. Body Soc.

[6] Jones DW, Woodbridge RS (2011).

[7] Bukman AJ, Teuscher D, Feskens EJ, van Baak MA, Meershoek A, Renes RJ (2014). Perceptions on healthy eating, physical activity and lifestyle advice: opportunities for adapting lifestyle interventions to individuals with low socioeconomic status. BMC Public Health.

[8] Zorbas C, Palermo C, Chung A, Iguacel I, Peeters A, Proietto J (2018). Factors perceived to influence healthy eating: a systematic review and meta-ethnographic synthesis of the literature. Nutr Res.

[9] Kelly S, Martin S, Kuhn I, Cowan A, Brayne C, Lafortune L (2016). Barriers and facilitators to the uptake and maintenance of healthy behaviours by people at mid-life: a rapid systematic review. PLoS One.

[10] Movimento Sem Terra (MST) (2023). Organic rice from MST crops: agroecology can produce on a large scale and oppose agribusiness. Movimento Sem Terra.

[11] Hallal PC, Lee IM, Sarmiento OL, Powell KE (2024). The future of physical activity: from sick individuals to healthy populations. Int J Epidemiol.

